# MinT: Middleware for Cooperative Interaction of Things

**DOI:** 10.3390/s17061452

**Published:** 2017-06-20

**Authors:** Soobin Jeon, Inbum Jung

**Affiliations:** Department of Computer Information and Communication Engineering, Kangwon National University, Chuncheon, Gangwondo 200-701, Korea; sbjeon@kangwon.ac.kr

**Keywords:** Internet of Things (IoT), middleware, distributed, performance

## Abstract

This paper proposes an Internet of Things (IoT) middleware called Middleware for Cooperative Interaction of Things (MinT). MinT supports a fully distributed IoT environment in which IoT devices directly connect to peripheral devices easily construct a local or global network, and share their data in an energy efficient manner. MinT provides a sensor abstract layer, a system layer and an interaction layer. These enable integrated sensing device operations, efficient resource management, and active interconnection between peripheral IoT devices. In addition, MinT provides a high-level API to develop IoT devices easily for IoT device developers. We aim to enhance the energy efficiency and performance of IoT devices through the performance improvements offered by MinT resource management and request processing. The experimental results show that the average request rate increased by 25% compared to Californium, which is a middleware for efficient interaction in IoT environments with powerful performance, an average response time decrease of 90% when resource management was used, and power consumption decreased by up to 68%. Finally, the proposed platform can reduce the latency and power consumption of IoT devices.

## 1. Introduction

The Internet of Things (IoT) provides information services for everyday use, and is built upon interconnections between sensor devices and network platforms. The IoT operates by receiving and integrating information from many interconnected devices to provide information-related services [1,2].

Most existing IoT middlewares have been focused on a WSN or cloud system base. A WSN mainly uses resource-constrained devices such as battery-based ones, or ones that have low-performance or low-storage. The WSN comprises a variable number of low-power nodes that communicate with each other, and work together in a particular region, to monitor different environmental variables measured by sensors included in the nodes. In addition, a base station, central node, gateway, or sink gathers the data from other nodes to analyze the data in the network environment. In a WSN, a node cannot self-operate or interconnect and interact with peripheral nodes to become an IoT device; this is because its role is solely to transmit data to the central system. WSN nodes are isolated, and devices cannot take part in other activities. Performing the role of an IoT device in this environment can therefore result in increased costs and reduced functionality [3].

The IoT allows for communication among different IoT devices through the use of the internet, at any time and in any location. IoT devices connected to each other can share their information efficiently, and cooperate with each other. However, when a unique server stores and manages all measured IoT device data—as is the case when using a WSN or cloud environment with a vertical structure—many problems arise during the process of sharing and using information, including network congestion, latency, and packet loss. The physically distributed IoT environment is therefore an essential IoT technology, in that it allows local IoT devices to share data and interact with each other. In distributed networks, IoT devices operate by themselves in a horizontal structure. They perform data processing and analysis functions that were previously carried out on a single server, and they connect to each other and share their data themselves. Distributed environments can therefore reduce the overhead that occurs when using a central server system such as a WSN or cloud-based network.

Recently, many examples of IoT middleware that can construct a distributed environment have been described. They can improve energy efficiency, data reliability and resource scalability, by interacting and operating between IoT devices within a small area or global network. Existing middleware based on distributed environment aims to improve the integrated management of heterogeneous devices, the integrated connection of heterogeneous networks, and the efficient discovery and usage of data. In addition, they provide a high-level API for use by device developers; this allows developers to control sensor devices, share sensor resources, and easily develop services [3,4]. However, since there is still a lack of research aimed at improving the energy efficiency and network reliability of IoT devices in local or global IoT environments, and additional studies are needed: on topics such as enhanced performance management for IoT device, efficient sensor devices, resource management and improved ease of development, etc.

In this paper, to allow construction of an efficient IoT environment, we propose a middleware for cooperative interaction of things (MinT). The MinT is a distributed middleware that aims to energy efficient processing and rapid response to requests. This allows efficient interaction between IoT devices, taking into account power consumption, network delay, and reliability. MinT provides all the basic features for operating a distributed IoT device. First, MinT provides a sensor abstract layer to allow easy development and operation of heterogeneous sensor and network devices. Second, it provides an interaction layer to support active interaction between IoT devices. Third, it provides a system layer to allow both a rapid response to requests from other IoT devices, and optimized resource management. The system layer can improve the performance and energy efficiency of an IoT device through efficient management of sensor devices and data. Using this layer, MinT can process requests and respond quickly, and ensures network scalability and reliability in a distributed IoT environment. Finally, IoT application or service developers require a high-level programming interface to develop sensor controls, resource sharing, and service applications. MinT therefore allows easy development by providing different types of interfaces which can operate all middleware features.

MinT provides an efficient way to handle messages, which enables fast responses on the IoT network. The performance of MinT is evaluated and compared with existing IoT middleware, focusing on the processing of requests in server test beds. The performance metrics used for carrying out assessments are the total throughput, and the throughput per packet block size. To investigate the efficiency of the sensor device management, energy efficiency and response times are also evaluated.

The rest of this paper is organized as follows: Section 2 describes the key requirements of IoT middleware. Section 3 describes all work related to existing IoT middleware. Section 4 describes the design and development of the MinT model proposed in this paper. Section 5 describes the implementation of MinT. In Section 6, the performance characteristics of the newly introduced IoT middleware are measured and evaluated. Section 7 concludes the paper.

## 2. Middleware Requirements

Recently, many IoT middleware solutions have been researched and developed. Middleware provides a software layer between applications, the operating system (OS), and the network communications layer, and coordinates some aspects of cooperative processing. Generally, middleware abstracts the complexities of the system or hardware, allowing application developers to develop devices and applications simply and easily. IoT middleware can ease the development process by integrating heterogeneous computing and communications devices, and by supporting interoperability within diverse applications and services. In particular, because an IoT environment can display considerable heterogeneity in both communication and system level technologies, IoT middleware should support both perspectives as necessary. IoT middleware must therefore integrate these technologies to support the potentially diverse application domains; namely, existing wireless sensor networks (WSNs), M2M (Machine to machine) communications, and supervisory control and data aggregation. As follows, a set of requirements for a middleware to support the IoT is outlined [2,3,5]:

*Physically Distributed***:** The IoT devices in a large-scale IoT system exchange information and collaborate with each other. They are likely to be geographically distributed. Most existing WSN or cloud system middleware is server-centric, and manages and operates all IoT devices connected to the internet. Because all data gathering, processing and service/application work is completed on a single unique server, information management and service or application deployment may be easily accomplished. However, because connections are directed towards one unique server, server problems can paralyze all services associated with that server. In addition, an IoT device often requests information from peripheral IoT devices; in a server-centric system, the IoT device must connect to a distant main server to get the information for a nearby peripheral IoT device, which can lead to unnecessary connections and power consumption. Peripheral IoT device is other IoT device in the same place as IoT device that attempts to make a request to the server. Because middleware must support functions that are distributed across the physical infrastructure of the IoT, a centralized view or middleware implementation will therefore be insufficient to support many distributed services or applications, a middleware implementation needs to support functions that are distributed across the physical infrastructure of the IoT. In distributed IoT environment, IoT devices themselves perform most roles (i.e., data processing, analyzing and sharing), normally operated by a server. A distributed environment can therefore reduce the overhead that occurs in centralized server systems such as a WSN or cloud-based network.

*Heterogeneous Devices*: Various IoT hardware platforms have recently been researched and developed; these range from constrained low-performance devices, which include an 8-bit multi-point control unit, to high-performance devices, which include 64-bit multi-core computer processing units (CPUs) and run on common operating systems. To minimize power consumption and the impact of such devices on the environment, low-power radios are likely to be used for connecting to the Internet; these radios do not use Wi-Fi or well-established cellular network technologies. However, the IoT is composed not only of embedded devices and sensors, but also of higher-order computing devices required to perform heavier duty tasks (for example, routing, switching, or data processing). Device heterogeneity emerges both from differences in capacity and features, and from other reasons such as multi-vendor product and application requirements. Because heterogeneous devices have different methods of connection, usage, and development, a device developer requires considerable time to develop an IoT device. For rapid development of heterogeneous devices, there is therefore a need to provide IoT device developers with simplicity, convenience and the ability to reuse code.

*Resource Management and Discovery*: Resource management is one of the key features of IoT devices. An IoT environment always manages resources that measure data from sensor devices, or services data from other IoT devices. IoT middleware must provide full resource management, including data gathering, processing and storage. In addition, efficient advertising and discovery of resources is required; these are measured and provided by IoT devices. Most IoT middleware fulfils a role by which a unique server manages all resources and all IoT devices connected to the internet. The managed resources can be easily discovered and provided by the main server; however, this method has drawbacks including server overhead, processing delays, network congestion, and so on. For efficient interaction between IoT devices, each device that is not a central server must be able to manage and share its own resources.

*Spontaneous Interaction*: IoT will integrate devices, many of which will be mobile, wirelessly connected, and resource constrained. Mobile IoT devices within the network leave or join anytime they want. Also, IoT devices can be disconnected due to poor wireless links or battery shortage. These factors will make the network in IoT highly dynamic. Therefore, IoT devices will need to cooperate to keep the network connected and active. In IoT environment, sudden interactions can take place as IoT devices move around, and come into other IoT device’s communication range, leading to the spontaneous generation of events. For example, a smartphone user can come in close contact with a TV/fridge/washing machine at home and that can generate events without the user’s involvement. Typically, in IoT, an interaction with an object means that an event is generated and is pushed to the system without much human attention.

*Large-scale network and large number of events*: In an IoT environment, thousands of devices or things may interact with each other, even in a single location (for example, a building, supermarket, or university); this is at a much larger scale than most conventional networking systems. An IoT device is configured with various IoT hardware platforms, ranging from constrained low-performance devices to high-performance devices. Each IoT device interacts with others periodically to request, respond or share their information. If IoT devices process received requests late and message transmission is sequentially delayed, then responses to requests cannot be sent on time. The processing delays impact all connected IoT devices, and the entire information exchange chain can be severely delayed; this may in turn cause network latency, a reduction in reliability, network congestion, and so on. IoT middleware must therefore provide scalability, reliability and availability to an IoT network, through rapid processing within IoT devices.

*Programming abstraction and ease-of-deployment*: Providing an API for developers is an important functional requirement for any IoT middleware. High-level programming interfaces must isolate the application or device development from the operations provided by the underlying, heterogeneous sensing or network devices. The level of abstraction, programming paradigm, and interface type must all be considered when defining an API. The level of abstraction refers to how the application developer views the system (for example, at the individual node or device level, or the system level). The programming paradigm deals with the model for developing or programming applications or services. The interface type defines the style of the programming interface. In addition, because a user typically deploys IoT middleware, deployment should not require expert knowledge or support; complicated installation and setup procedures must be avoided. 

## 3. Related Works

Recently, a number of OSs have been developed to support the development of IoT middleware solutions. These include Contiki, Brillo, mbed, RIOT, FreeRTOS, Embedded Linux, OpenWSN, TinyOS and Tizen [6,7,8,9,10,11,12,13,14]. However, existing middleware based on the above OSs cannot support the requirements of most IoT environments as mentioned in Section 2. In this section, we therefore describe all work related to existing IoT middleware; namely, WSN and cloud-centric IoT middleware, and distributed IoT middleware based on the requirements mentioned in Section 2. In the interest of space, the discussion of each work highlights only key points, without exhaustively capturing its performance against all requirements. Table 1 is the comparative table to summize of the middlewares.

*WSN and Cloud-centric IoT Middlewares***:** Hydra [15] is a type of middleware used for ambient intelligence services and systems. Hydra provides semantic level interoperability using semantic web-based technology. Hydra’s resource, device, and policy managers make it lightweight, by optimizing power consumption in resource-constrained devices. However, ontology-based semantic interoperability solutions implemented in Hydra are likely to be unsuitable in IoT, because there are currently no standard ontologies for ultra-large-scale IoTs. UbiSoAP [16] provides seamless networking of web services. UbiSoAP is a lightweight middleware solution, which offers network level interoperability by supporting heterogeneous networking devices and technologies. However, ubiSOAP cannot be operated by itself, and this is key for the adaptive and autonomous behavior of the things. The mobile sensor data processing engine (MOSDEN) [17] is a very similar WSN-centric middleware for IoT environments. It supports sensing as a service model [18], built on top of Global Sensor Networks (GSN). The use of a plugin architecture improves the scalability and user friendliness of the middleware; this is because plugins for heterogeneous devices are easier to build and available in easily accessible places. An information of IoT devices, which include sensor devices, is always updated in the IoT environment, because the environment and the state of the device may change periodically. However, MOSDEN may suffer in an IoT environment because it predefines the composition rules available in the virtual sensors, and this may not work well in the dynamic and large networks of an IoT. 

Many cloud-based IoT platforms are also available. Xively [19] is a PaaS that provides middleware services to allow creation of products and solutions for IoT. It offers developers a standards-based directory, data, and business service. Its web-based tools simplify data and control other complexities of IoT application development, and it also supports multiple data formats. However, it does not homogenize the incoming data, so data processing must be either completed individually for each source, or standardized using a prior mapping process. The standardized incoming data refers to the configuration of the protocol standards that can collectively recognize multiple data formats. This creates an overhead in the system. CarrIoTs [20] is a cloud-based platform for IoT environments. It focuses particularly on M2M communications, cost effective M2M application development, scalability, and ease of use; however, the main advantage of CarrIoTs is that it supports network level scalability. Both CarrIoTs and Xively benefit from active online communities and are popular among developers and new adopters. However, like Xively, CarrIoTs does not standardize the incoming data. 

ThingSpeak is an open application platform which is designed to enable meaningful connections between things and people. It provides developers with APIs to store and retrieve data from sensors and devices using HTTP over the Internet. It has real-time data collection, processing and data visualization. ThingSpeak has Write API Key to update channel. Each Channel of ThingSpeak supports data entries of up to eight data fields, latitude, longitude, elevation, and status [21,22]. The WSN or cloud systems based on a central server provide ease of information gathering, processing, and deployment. However, they also cause many problems (i.e., network congestion and delay, transmission loss, and so on) in IoT environments in which IoT devices frequently interact with each other to obtain peripheral IoT device information. In addition, most of these examples of middleware do not allow IoT devices to independently interact and operate with each other.

*Non-server based Distributed IoT Middlewares*: IoTivity is an open source IoT middleware solution that has been adopted as the standard middleware technology by the Open Interconnect Consortium. The consortium is organized by Samsung Electronics, Intel, Cisco, and GE, among others. IoTivity consists of four modules, as follows: The Discovery module, which is responsible for finding local and/or remote devices; the Data Transmission module, for exchanging and controlling information; the Device Management module, for managing device configurations and privileges; and the Data Management module, which implements data collection and analysis. Notably, IoTivity supports thread pooling to process requests simultaneously. Because IoTivity works on the Constrained Application Protocol (CoAP), the platform can easily support devices with low specifications and low power. However, because the IoTivity device control mechanism is complex, considerable time and effort would be required for installation and development of the experimental platform. To develop an IoT device, the process that connects the sensor and network devices to the hardware platform is required. However, IoT device developer with IoTivity need to work on additional tasks that are sensor porting, driver development and so on to connect sensor or network devices to hardware platform because IoTivity do not support above process for developing IoT device [23].

Android Things is an IoT platform developed by Google, and is based on the kernel and upper subsystems of the Android operating system. Android Things is aimed at cross-platform applications for IoT devices with low power and low hardware specifications. It supports cloud systems based on M2M (Machine to machine) communication methods [7]. Californium is an open source IoT platform based on the CoAP, and developed using Java. It is composed of: a Network State module, which supports connections through various network protocols; a Protocol Stage module, which processes request messages; and the Business Logic State, which provides services to users. In addition, because Californium simplifies the Protocol Stage and introduces multi-threading, IoT systems operating on this platform can provide quick responses to multiple requests within a short timeframe [24]. nCoAP is an open source IoT communication platform based on the CoAP, which uses a Netty framework to support non-block asynchronous communication. Within this framework, nCoAP uses a multi-threading model with thread pooling to process requests simultaneously and quickly [25].

In Section 2 and Section 3, we recognized the requirements of IoT middleware, and analyzed existing IoT middleware. Notably, Distributed IoT middleware can improve energy efficiency, data reliability and resource scalability by interacting with and operating between IoT devices, within a small area or global network. However, the problems exist within existing IoT middleware, which must be addressed to allow operation of IoT devices in distributed environments. 

First, the ease of development of heterogeneous sensor and network devices must be improved. Most existing IoT middleware does not aim to provide high-level programming interfaces for the development of sensor or network devices. In addition, IoT middlewares, which provide the API for IoT device development, do not provide ease of development, such as reusability and simplicity of development.

Second, IoT devices must efficient process requests from other IoT devices. In an IoT environment, thousands of devices or things may interact with each other, even in a single location (for example, a building, supermarket, or university). However, if IoT devices are late in processing the received requests, and message transmission is sequentially delayed, the responses to requests cannot be sent on time. These processing delays impact all connected IoT devices, and the entire chain of information exchange can result in network latency and packet loss. This can affect the reliability of information exchange in IoT networks.

Third, efficient management of sensor devices and resources is required. An IoT device uses its own sensor to measure the data requested from other IoT devices. The sensor takes a certain amount of time to measure the data, and because an IoT device must wait for the sensor device’s response, there is a delay when responding to a data request. In addition, there is a lack of connectivity between the sensor device management, which operates the sensor device, and the interaction management, which processes the requests from other IoT devices. The IoT device measures the data using its sensor, and provides measured data to other connected IoT devices; however, because both are operated separately, slight delays and unnecessary power consumption result.

In this paper, we propose improved middleware that both satisfies the requirements of IoT middleware, and solves the problems of the existing middleware solutions. This new IoT middleware will allow more efficient development and operation of IoT devices.

## 4. Middleware for Cooperative Interaction of Things

In this paper, we propose an IoT middleware solution called MinT, which is aimed at supporting the cooperative interaction of things. The solution can operate IoT devices in a fully distributed IoT network, and adapts to IoT environments by recognizing the requirements of IoT middleware. To satisfy the requirements of IoT middleware, the goals of MinT are as follows:

- Support the hardware abstraction layer to allow development of various sensors and network devices.
- Provide a network environment to support active interaction between IoT devices.
- Improve processing performance and network reliability.
- Allow energy-efficient sensor device and data management.
- Enable easy installation, and provide a simple high-level API.

**Figure 1:**
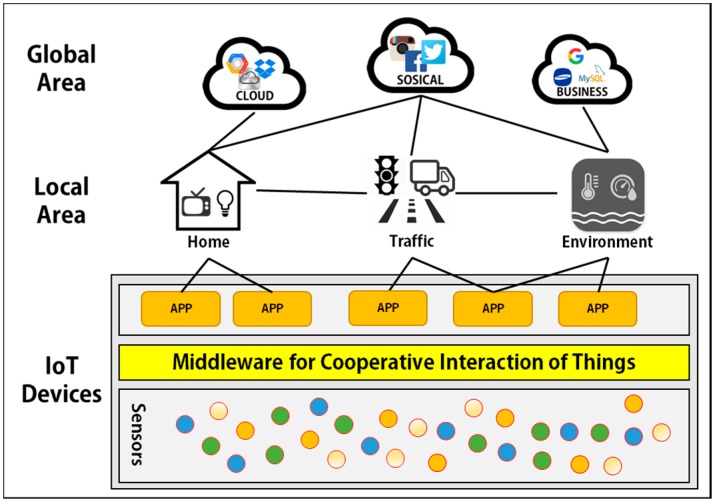
IoT overview based on the proposed middleware.

First, MinT supports a hardware abstract layer called sensor abstract layer (SAL) to allow development of heterogeneous sensor and network devices. Figure 1 shows an overview of an IoT working environment as implemented using MinT middleware. The function of an IoT device is to manage and operate connected devices in the IoT. The sensors and network devices work together to collect local information, and then deliver this information to other IoT devices or environments. MinT middleware provides a sensor abstract layer (SAL) for various sensors or network devices. The SAL aids in effectively developing and operating sensors and network devices as a system. MinT middleware provides a platform interface to support Linux, Android and Windows platforms. In addition, it provides interfaces such as GPIO, ADC, SPI, and UART to allow connection of various types of hardware, including the Beagle Bone Series, Raspberry Pi Series, and the Edison with multiple sensor types. These interfaces aid in the rapid and convenient development of IoT devices. 

Second, as shown in Figure 1, an IoT device implemented by MinT can interact spontaneously with peripheral IoT devices connected in the same region and network area. For this purpose, MinT provides the following features.

MinT provides a network environment to support active interactions between heterogeneous network protocols. An IoT environment supports various network protocols such as Bluetooth, BLE, ZigBee, and Wi-Fi; when a united network environment is installed, connectivity can be established between IoT devices via any of the protocols, based on the quality of the physical network connection and flexible use of system resources. MinT middleware supports Bluetooth, BLE, ZigBee, and IP based protocol to connect IoT devices via various network protocols. In addition, taking into consideration the specifications of IoT environments, MinT uses the CoAP [26] and MQTT [27] application protocols. Both are IoT application protocols that can be used in both cloud and distributed service environments. They can enable efficient resource exchange between both high-performance IoT devices and low-power constrained IoT devices.

In addition, MinT provides a system process model to improve processing performance and network reliability. IoT networks can be configured with numerous devices, ranging in size from local area to global area networks. In distributed networks, rapid request processing is one of the most important factors in IoT environments. Because processing delays can cause service and network latency across the entire network, IoT devices require efficient and rapid processing within the middleware. To improve processing performance, MinT prioritizes system resource utilization towards the Interaction Layer; this layer manages and processes requests received from other IoT devices. MinT also supports multi-threading in the Interaction Layer, to allow efficient processing of requests according to the hardware platform performance.

Third, to allow energy-efficient sensor device and data management, a method for specifically managing the sensor device and data is required. IoT devices provide various services to users or other IoT devices using installed sensor devices; they may also perform certain activities using data that is either obtained from sensors installed on their own or received from other IoT devices. Generally, the data not managed by middleware such like caching policy is deleted immediately after use. If IoT device wants to use same data that have been deleted before, it must activate its sensor device or send a request to another IoT device to get same data again.

Most IoT middleware supports a network-caching policy to leverage data from other IoT devices. Network-caching policy that reuses received data has the advantage of reducing network throughput and energy consumption. IoT device obtains data from its sensors and provides its data to other IoT devices. Existing middlewares provide default module to operate installed sensor in IoT device. However, they activate the sensors of IoT device to obtain sensing data whenever there is a data request from other IoT device because they do not support how to control sensor devices and manage the data obtained from the sensors. It does not cause problems if there are few requests for a data. However, if more data requests are made, this can cause a processing delay, and can waste the energy of the IoT device because IoT device consumes a lot of energy for sensor operation and has a delay to acquire data from the sensor.

Therefore, to reduce unnecessary IoT device processing, a method for specifically managing the data used is required. MinT can manage the data measured by the sensor devices. The Resource Management defines two sensor measurement methods aimed at reducing unnecessary sensor device behavior. This method allows MinT to both enhance the efficiency of use of the sensor device, and reduce power consumption and processing delays. The Resource Manager can also manage the data received from other IoT devices for reuse.

Finally, MinT provides powerful development tools for service developers and clients. Java is used to support development on various platforms. By using these tools, users can develop new IoT devices and service applications that may be either standalone in nature, or may cooperate with other services to implement a new service. Because the MinT platform is easy to learn and has a user-friendly interface, both expert developers and general users can rapidly develop high-level business ideas from concept to implementation in IoT environments. As shown in Figure 1, the reduced development time enhances the ability of organizations to implement IoT systems with the latest sensor devices and service applications.

### 4.1. MinT Architecture

**Figure 2:**
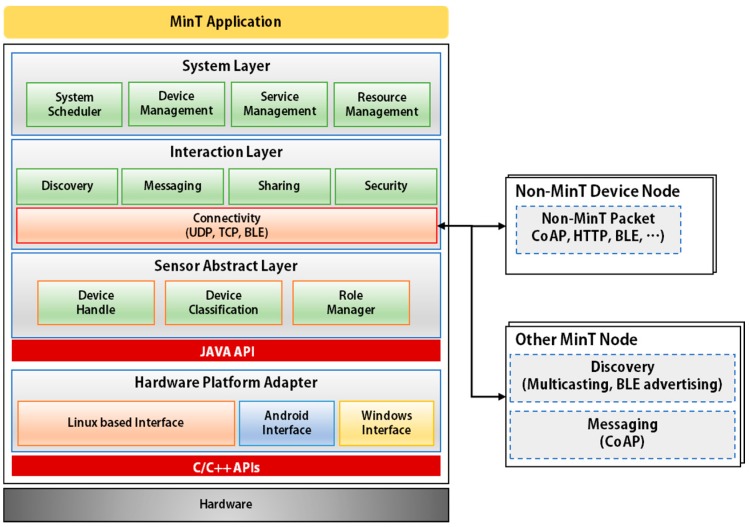
Architecture of MinT.

Figure 2 shows the architecture of MinT. The MinT application is composed of five layers: the Platform Adapter, Sensor Abstract Layer, Interaction Layer, System Layer, and Application Layer. 

The Hardware Platform Adapter provides the immediate upper interface to control the hardware installed as part of the IoT device. The Hardware Platform Adapter provides interfaces to the underlying hardware that let users control the sensors and network devices connected to the hardware platform. Support is provided via Linux-, Android-, and Windows-based interfaces.

The SAL abstracts the sensors and network devices connected to the platform. By way of this abstraction, they can be used easily and have their respective functions controlled. Each device is controlled via the device handler connected to the underlying Hardware Platform Adapter. The sensors classified from the SAL are managed by the Device management module of the System Layer. In addition, MinT connects the resource data stored and managed in Resource Management with sensor device according to role. Its role is defined by data collection and sensor operation. Role Manager is connected with Resource Management and assigns the role of each device.

The Interaction Layer is responsible for implementing all functionality related to network connections in the MinT middleware. Basically, it supports various network protocols such as UDP, TCP, BLE, Bluetooth, and ZigBee. The Interaction Layer provides functions related to Discovery, Messaging, Sharing, Security, and Connectivity. The Discovery module executes any needed searches of other objects or nodes by utilizing Multicasting on UDP and Adverting on BLE. Connectivity to non-MinT device nodes is established using protocols such as HTTP and DTLS.

The System Layer handles all the functions related to System scheduling, Device Management, Resource Management, and Service Management. The System Scheduler decides the priority of each manager to use CPU resource efficiently. 

The Application Layer provides APIs for developers and general users. By using these APIs, service applications in MinT can be programmed easily.

### 4.2. Hardware Platform Adapter (HPA) and Sensor Abstract Layer (SAL)

The MinT introduces the SAL for sensor devices. This approach provides for easy installation and convenient management of devices. Based on the HPA and SAL, the MinT is able to abstract the operation for devices from various manufacturers and provide a common interface for developing device drivers while enabling high levels of reusability.

**Figure 3:**
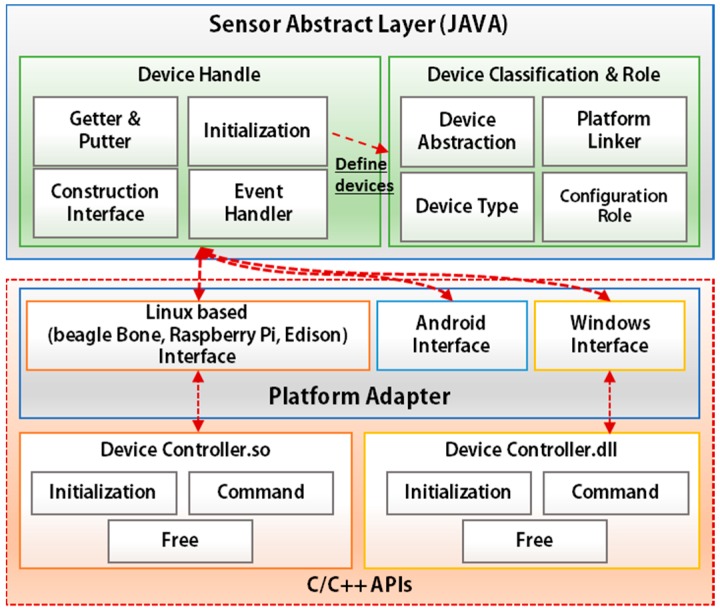
Sensor abstract layer architecture.

Figure 3 shows the architecture of HPA and SAL. The HPA provides the interface to control the platform. This interface is used for developing the IoT services that in turn control the devices. MinT supports I/O functions for various platforms including Linux, Android, and Windows. For each OS, the PA supports multiple interfaces including GPIO, ADC, SPI, UART, PWM, and I2C. 

The HPA is built on a comprehensive set of C/C++ APIs, which have effective tools to control embedded systems. To develop the device driver via HPA, we can refer to the HPA interface provided for each platform as shown in Table 2. Each interface provides a robust suite of all the tools that are necessary (initialization tool, command tool, etc.). As shown in Table 2, there are three functions that must be made available for device driver development. Once driver software is produced through the interfaces, the MinT builds and creates dynamic libraries (such as .so, .dll, and .jar) for each platform. The Device Handler of the SAL performs the function of abstracting to drivers on the basis of the referred interfaces. The Device Handler is constructed as follows:

- The initialization module refers to the Device Classification & Role module and defines the device type, role, and platform for the connected device drivers.
- The Getter & Putter module is used to build input/output functions.
- The Event Handler module controls asynchronous connections between the device driver and MinT.
- The Construction Interface provides the interfaces needed to connect the developed device driver with the HPA. The HPA, along with the CI, connects the device driver via JNI with the SAL.

The abstracting device drivers on SAL are managed by the Device Management module of the System Layer. The device driver implemented once on a particular platform can be reused on other platforms with the aid of the linking and rebuilding functionality provided in MinT.

### 4.3. Interaction Layer (IL)

**Figure 4:**
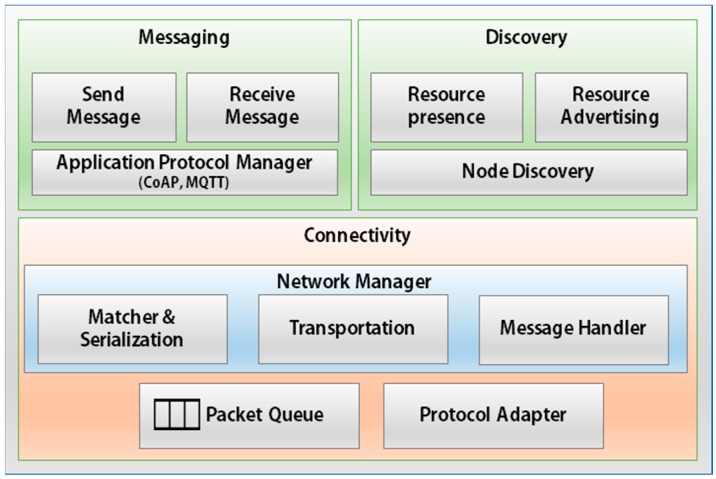
Interaction layer architecture.

Figure 4 shows the structure of the Interaction Layer. The key goals of the Interaction Layer are:

- Manage the process of creating connections for various network protocols.
- Improve the message reliability.
- Perform fast searches for surrounding devices and enable efficient allocation of resources.

The Connectivity module in MinT is responsible for supporting networking and connectivity between IoT devices. This module provides the Network Manager for efficient exchange of data between IoT devices. As shown in Figure 4, the Network Manager is composed of submodules in Matcher&Serialization, Transportation, and Message Handler that undertake all the operations related to data receiving/processing/sending. It is the Network Manager that is responsible for initiating, maintaining, and terminating network communications with other IoT device implemented with MinT and non-MinT devices via the Protocol Adapter. The Protocol Adapter abstracts all the network regulations for each protocol within it, which permits MinT to support various network protocols such as Bluetooth, BLE, Wi-Fi, and ZigBee. 

The Messaging module creates and analyzes packets based on application protocol. This module can be easily extended to support other application protocols such as CoAP or MQTT. MinT already supports the CoAP and MQTT in it. Generally, MinT uses CoAP for M2M communication between IoT devices. The Discovery module uses CoAP for searching the peripheral IoT devices.

#### 4.3.1. Connectivity Module

**Figure 5:**
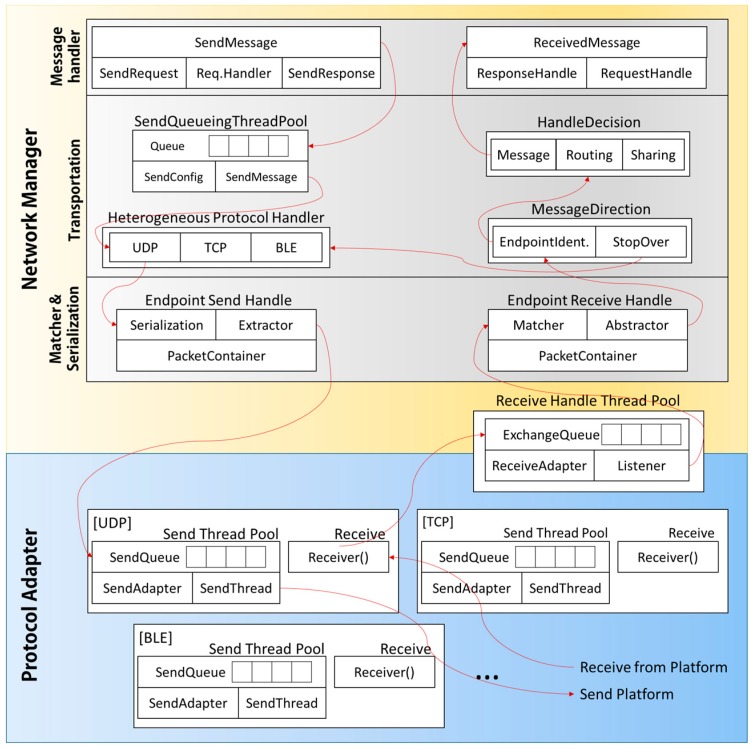
Connectivity module structure and flow.

As shown in Figure 5, the Connectivity Module is composed of the Protocol Adapter and Network Manager. The Protocol Adapter encapsulates the definitions for all the network protocols supported by MinT. It physically connects to real network devices through a Device Management module. The Protocol Adapter abstracts the Send and Receive modules for data sending/receiving operations. These abstracted modules are redefined according to each protocol. 

The Send module creates thread pools for sending data according to the requirements of each protocol. The data to be sent is stored in the SendQueue and the Send Task thread performs the actual sending of data.

One Receive module is assigned for each protocol, which receives and processes the data received from the outside. All the data processed by the Receive module is stored into the Exchange-Queue of the corresponding Receive-Handler-Thread-Pool (RHTP). Given the fact that the data received for all supported protocols should be processed in the Network Manager, all the receiving procedures are managed on a single RHTP. The received data stored in the Exchange-Queue are processed in the Network Manager. 

RHTP processes the received data/message stored in Exchange-Queue through Network Manager. The message requested by other IoT device must be responded to quickly. Therefore, RHTP, which is responsible for processing the requested message, occupies the most CPU resources in the MinT. RHTP adjusts the size of thread pool according to hardware platforms. Generally, The thread pool size is set up according to the number of CPU cores on each platform.

As seen in Figure 5, the Network Manager is composed of Matcher&Serialization (M&S), Transportation, and Message Handler (MH) modules. Each module has a specific role in the processing of the sent/received data. First, the M&S module analyses the packets from the received data present on the Exchange Queue. The analyzed packets are transforms into the data format for using it in MinT. After that, the packets are transferred to the Transportation module, which confirms their final destination. If the final destination of the packets is not the current object, the packets are re-transferred to the next destination. However, if the current object is indeed the final destination of the packet, the Handle Decision module analyses the purpose of the packets received. Based on the purpose of the analyzed packets, they are distributed to the Message Handler, Routing Handle, and Sharing Handle sub-modules. The Message Handler sub-module analyses the state of the transferred packets and according to it, they are delivered to the System Layer. 

If MinT or its users want to initiate a transfer of the data, they should utilize the SendMessage method for the Message Handler sub-module. The SendMessage method is classified as Request data or Response data according to the purpose. The results from SendMessage are stored into the Send Queue thread pool associated with the Transport module and the system waits for the transmission to complete. The Transportation module uses the Heterogeneous protocol handler to decide the protocol to be used for the SendMessage method. Finally, the M&S module creates the corresponding handle to transfer the SendMessage and delivers it to the Protocol Adapter. The Protocol Adapter begins to transfer the data to each destination. Figure 5 shows the flows for the data send/receive operations. 

#### 4.3.2. Messaging and Discovery Module

**Figure 6:**
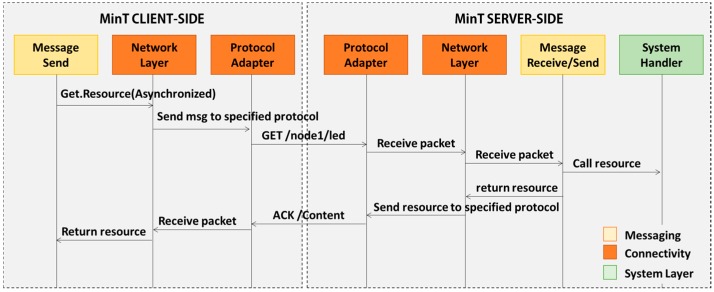
Messaging sequence diagram on MinT.

Figure 6 shows the basic messaging sequence diagram used in MinT. The Send Message and Receive modules are used to accomplish transmission of data. These two modules are inherited from the Message module. The Message module is classified as the Request and Response data according to the purpose of packets. As shown in Figure 6, the request data in the client is included in to GET, POST, PUT, and DELETE messages that are RESTful API [26] and sent to the server. The server that received request data from the client send the response data to client corresponding to the request data.

In Figure 6, when the client uses the Send Message functionality to request resources, the GET message is transferred to the server. This message is delivered via the Network Manager and Protocol Adapter of the Connectivity Module. The server analyses the received packet and uses the System Handler to search for the requested resource. The located resource is transferred to the client through the Connectivity Module. The client receives the requested resource in blocking or asynchronous mode.

**Figure 7:**
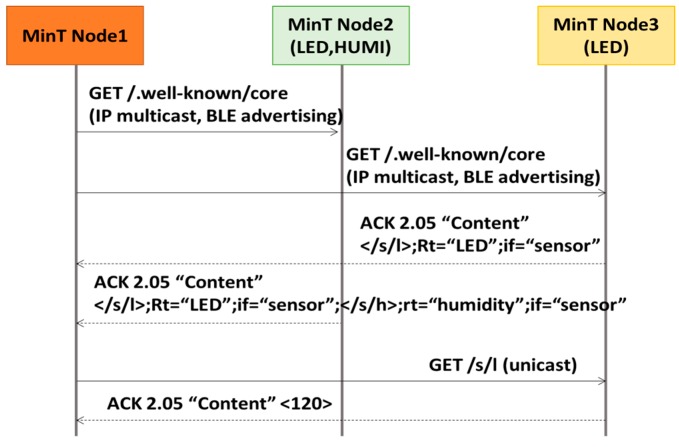
Sequence diagram for resource discovery and resource request in CoAP.

In the IoT environment, each IoT device is searching for information from its peripheral IoT devices. The Discovery module uses IP Multicast or Bluetooth (2.0, EDR, BLE) Advertise to search the peripheral IoT devices and retrieve the needed information. Figure 7 shows the sequence diagram for Resource Discovery and Resource Request in MinT. In order to search the surrounding IoT devices, the Node 1 sends a CoAP discovery (well-known/core) message to peripheral the IoT devices (Node 2 and 3) in question. According to the network protocol type, IP Multicast or Bluetooth Advertise is used for sending the message. Upon receiving the Discovery message, Node 2 and 3 in question returns their resource information as the response message. The Node 1 that receives the response message saves the resource information into its resource storage. The Node 1 on MinT uses the GET message in Unicast mode in order to retrieve the resource information from the resource storage of the target IoT devices. 

### 4.4. System Layer Structure

**Figure 8:**
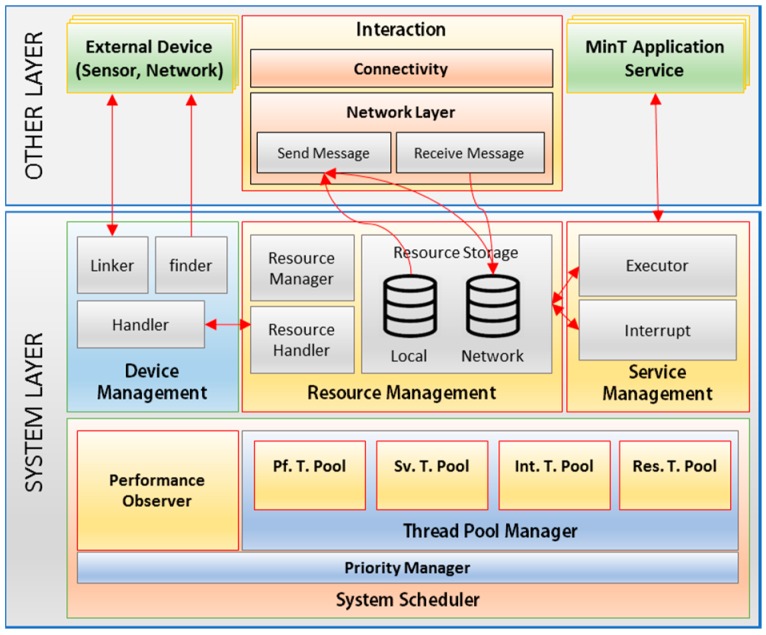
System layer structure.

Figure 8 shows the structure of the System layer in MinT. The System Layer’s functionality is implemented as multiple modules; namely, Device Management, Resource Management, Service Management, and System Scheduler. This allows the implementation of efficient system operations.

*Device Management*: By employing finder and linker sub-modules, the Device Management module can find or link to the sensing and networking-related device drivers connected to MinT. The Handler sub-module operates all linked sensor devices and manages the data with the Resource Handler sub-module of the Resource Management module. For example, Handler sub-module activates sensor device to gather sensing data and reports gathered data to the Resource Handler. The Resource Handler checks received data and stores it into the Local Storage of the Resource Management module. 

*Resource Management module*: This module is responsible for managing all local and network resources in the MinT middleware. The resources can be saved into local or network (connectivity) storage, as shown in Figure 8. To increase the reusability of data and network reliability, local storage stores sensing data from installed sensor device and connectivity storage stores the data received from external IoT devices. Both two storage are operated with a CoAP caching policy called Max-Age. This option indicates the maximum time a response may be cached before it is considered not fresh. A default value of three seconds is assumed to keep the received resources. If the data in the storage is not updated for three seconds, it is deleted. In addition, they use least recently used (LRU) algorithm for cache replacement, when the storage space is full [26].

The Resource Handler module cooperates with the Device Handler directly. Resource Management module provides two modules for operating sensor devices. The Thing Instruction module activates a sensor device according to requests received from another IoT device. The sensor device performs the requested operation through the Thing Instruction module.

**Figure 9:**
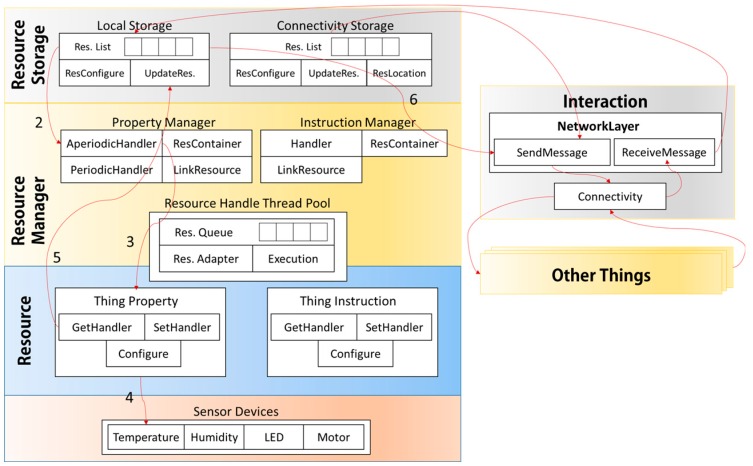
Resource management layer structure.

The Thing property is used to obtain measured data from the sensor device. As shown Figure 9, the operations used to gather a sensor device’s data are divided into aperiodic and periodic. An aperiodic method operates the sensor device, when IoT device receives the request from other IoT device or user. 

A periodic method periodically operates the sensor device and stores measured data into local storage. An IoT device, which provides the sensing data to other IoT device, can receive a large amount of requests from large number of IoT devices. However, if IoT device operates the sensor device to measure the sensing data, which are less variation, according to amount of requests, it can consume unnecessary energy.

Therefore, when an IoT device measures and stores data into local storage according to the degree by which the data has changed Figure 9(5) and then supplies data to application or other IoT device with the data Figure 9(6), this increases the energy efficiency of the IoT device. MinT provides an approach that can adjust the measurement period of a sensor device according to the characteristics of the device and the level of data requests. We will also evaluate the processing time and power consumption through experiments. Currently, IoT device developer analyzes the characteristics of the device and environment and operates the adjustment of the sensing period. In the future work, we need to study the active adjustment system of sensing period according to the characteristics of the device and the level of data requests.

*System Scheduler*: This is an integrated thread management tool, which handles threads created from the System Layer, Interaction Layer, and Application Layer. As shown in Figure 8, the System Scheduler is composed of the Thread Pool Manager, Priority Manager, and Performance Observer. The Thread Pool Manager manages four distinct types of thread pool. The maximum size of all thread pool is assigned to be equal to the number of CPU cores. 

First, Service Thread Pool (SV. T. Pool) is used to support the complete group of services used in MinT, in the Service Management module of the System Layer. Given the fact that the number of threads created in each application is different, and that all services cannot work simultaneously. Second, Resource Thread Pool (Res. T. Pool) supports resource handling for the resources collected from external sensing devices. Third, Interaction Thread Pool (Int. T. Pool) is used in the Interaction Layer, and is the thread pool management group for the Protocol Adapter and Send/Receive Handler. The size of the waiting queue is set to be between 1 KB and 10 MB, according to the size of the platform memory. Fourth, Performance Thread Pool (Pf. T. Pool) manages and measures the performance of each thread pool group, and is operated in conjunction with the Performance Observer in the System Layer. A separate thread is created for each thread pool to measure its performance.

*Service Management module*: This module is primarily utilized during the development of application services. The APIs built into MinT enable the rapid building of IoT applications, with functionality such as control of deployed sensors, resource searching, resource release, and information requests.

### 4.5. Interaction between IoT Devices

**Figure 10:**
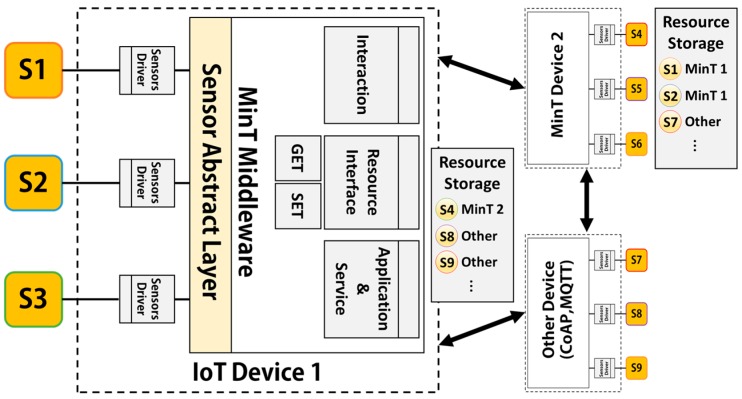
Interactions between IoT devices (MinT and other IoT deivce).

Figure 10 shows the IoT device implemented in MinT. MinT is connected to the sensor devices, and operates them through the SAL. Each sensor device can provide its own measured data to other IoT devices through association with the Resource Interface. MinT is distributed service middleware and therefore, to interact with peripheral IoT devices, it advertises itself and discovers peripheral IoT devices. In addition, it can interact and share with other IoT devices that are not implemented by MinT, because it supports various application protocols and RESTful structure for packet messaging as mentioned Section 4.3.2. MinT receives the response data and saves the received information into its resource storage; stored data can then be reused, and are updated according to scheduling rules. MinT also provides various services (providing peripheral information, activities, etc.) to users using the stored data.

In an IoT environment, at regular intervals each IoT device shares a considerable amount of information with the IoT devices in its surrounding area. To enable effective information sharing between local and remote IoT devices, MinT restricts the scope of the Discovery message.

## 5. Implementation

In this paper, MinT was implemented on C and JAVA. It is available to any developer with open source code at: http://sn.kangwon.ac.kr/LabThings/MinT. Currently, MinT supports hardware platforms, which include the Beagle Bone Series, Raspberry Pi Series, and the Edison, and operating systems including Linux, Windows and Android. It provides two types of development environments. The first is the development tool for device drivers to control the sensor or network device connected in a hardware platform. The second is the other one for IoT application developer to operate the IoT device with device driver and MinT framework. In this section, we describe each development environments and implement the IoT server device and client devices for performance experiment of MinT system.

### 5.1. Device Driver Development

The SAL proposed in this paper allows easy installation of a sensor and network device into an IoT device, and also simplifies further development. MinT provides an interface that helps to develop a device driver that abstracts the device of each manufacturer, and also enables reuse. In this section, we describe the development of example drivers that measure distance using an ultrasonic sensor. An IoT device can connect using an ultrasonic sensor with a UART connection.

**Figure 11:**
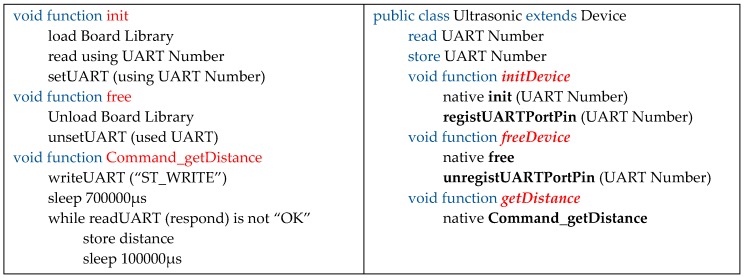
Device controller C (**left**) and device driver Java (**right**) development for ultrasonic sensors.

To develop a device driver for the ultrasonic sensor, the device controller must first be developed as shown in Figure 11 (left). The device controller is based on the C language to allow embedded system programming, and is developed by referring to the interface provided in Table 2, in combination with MinT. The Init function loads the device library and sets the UART number for connection of the ultrasonic sensor. The Free function disables the device library and frees unnecessary memory, such as that used for the UART connection. The Command function creates a general function to operate the sensor device; this function includes the getDistance function to obtain distance data from the ultrasonic sensor.

Because MinT is IoT middleware based on Java, the device driver should ultimately be provided in Java; this allows the association of SAL with MinT, as shown in Figure 11 (right). In order to develop a device driver, the MinT API Device class must be inherited; the device driver is then developed by referring to the device controller. Finally, the device controller and driver are linked by the java native interface (JNI) and the final device driver is created. A device driver implemented once on a particular hardware platform can be reused on other hardware platforms, with the aid of the linking and rebuilding functionality provided by MinT.

### 5.2. IoT Device Development

**Figure 12:**
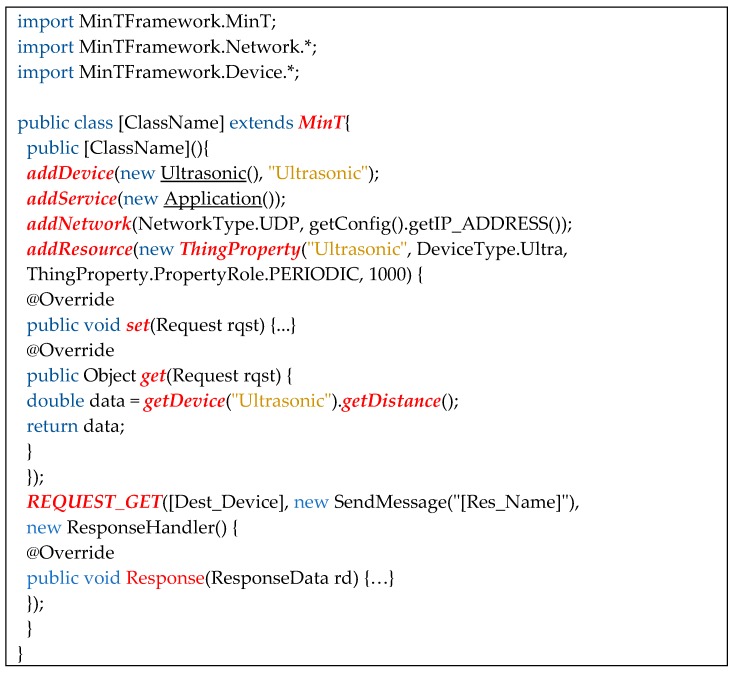
IoT device application development using MinT.

MinT aims to provide a simple and easy-to-use development kit for IoT device developers. This allows developers to develop IoT devices with minimal effort. Figure 12 shows source code used for IoT device application development, implemented in MinT. First, it inherits the MinT class and uses the MinT base middleware. MinT typically provides approximately five functions required to operate an IoT device.

The “addDevice” function can add the sensor devices created in Section 5.1. The “addservice” function adds the application class that defines the IoT device operations or services, while “addNetwork” adds network protocols (for example, UDP, TCP/IP, BLE and Zigbee). The “addResource” function can add a resource that is shared with peripheral IoT devices; to use this function, the resource name, resource type, and the resource update period described in Section 4.3.1 are set to the “addResource” attribute. When “addResource” is created in the main class, we can define the “set” and “get” functions. The “set” function defines the behavior of the sensor device; when an IoT device receives a request to operate the sensor device, it operates the sensor device associated with the set function in the requested resource. The “get” function can connect to a sensor device, and provides another IoT device with the measured sensor data. The “addResource” function in Figure 12 shows that an ultrasonic resource is added to the IoT device, and the measured distance from the ultrasonic device is returned by the “get” function in “addResource”.

Finally, to request information from another IoT device, the REQUEST function can be used. The REQUEST function includes GET, SET, PUT, POST, and DELETE functions. Figure 12 shows that the IoT device requests resource data [Resource Name] from another IoT device [Destination Device] using the REQUEST_GET function.

## 6. Performance Evaluation and Results

In this section we evaluate the power consumption, processing delay, reliability, and processing performance of MinT. In a typical IoT environment, several IoT devices are designed with embedded devices used for network communications. Many of these are typically dependent on limited power sources such as batteries for operation. Such devices must optimize the power consumed by the sensor device operation and message exchange. MinT allows an IoT developer to adjust the measurement cycle of a sensor device, which will in turn alter the power consumption. We therefore evaluate the power consumption during the experiments using the measurement cycle; we also evaluate processing delays resulting from resource management.

While information and requests may be flowing smoothly through large sections of an IoT network, it is possible that at a given point in time a very small number of IoT devices are suffering a degradation in performance due to network bottlenecks. This can result in a domino effect with the performance of other connected IoT devices. To address this issue, the middleware should enable quick and efficient processing for those IoT devices saturated with requests. Performance enhancement introduced by middleware can make a significant difference in maintaining overall IoT system-level performance. In this section, we experimentally evaluate the processing performance of MinT, using throughput and scalability as the performance metrics for comparison.

### 6.1. Experimental Environment

Basically, we use the CoAP protocol for experimentation and evaluation. Using the CoAP protocol, IoT devices, which are high-end or low-power constrained hardware platform, can share their resources efficiently each other. Therefore, most of IoT platforms support the CoAP protocol and use the main application protocol of them. In the basic test scenario, all of the IoT devices can sent verifiable response data (“hello world”) to simple GET requests (/request), and each of the request and response data records was assigned a CoAP header. In addition, we used the yardstick program structure, consisting of a load generator and virtual clients, in the Internet protocol to achieve accurate performance measurements [28].

**Figure 13:**
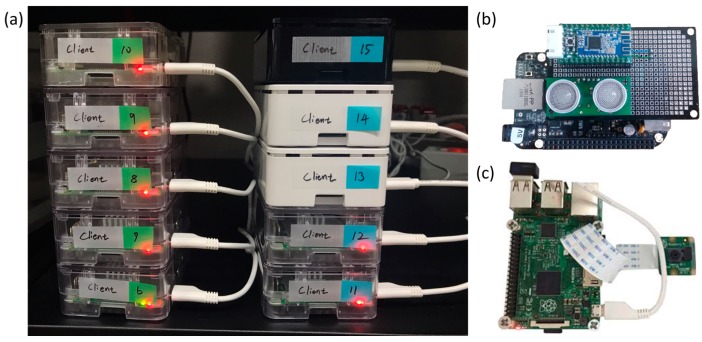
Experiment environment implemented using MinT. (**a**) client modules for experiment, (**b**) server module with ultrasonic sensor, (**c**) server module with camera sensor.

We construct experimental setup, with ten Raspberry Pi 3s for the virtual clients and four servers as shown in Figure 13a. The client modules are the IoT testbeds without sensor devices in order to send packets periodically to the server. The specifications of the client (Raspberry Pi 3) were: ARM Coretex-A53 CPU, 1 GB RAM, and Linux OS. Figure 13b,c show the IoT device servers implemented usig MinT. They are tested on hardware platforms including Beaglebone Black, Raspberry Pi 3 and Intel Edison. Each device installs the camera sensor, ultrasonic sensor and BLE module. The ultrasonic sensor was developed with a Devantech SRF-04 and its maximum detection range is up to 7 m. It therefore takes about 46 ms and to measure the distance from it [29]. The camera sensor was developed with a Rspberry Pi Foundation Raspberry Pi Cameara Module v2 [30] and it takes about 832 ms to take a picture. We also used BLE module with Jinan Huamao Technology (Jinan, China) HM-10 [31]. To connect between clients and servers, we used a gigabit Ethernet router with either wireless or wired connections to the server platforms.

With this test setup based on ten Raspberry Pi 3s, the load generator can vary the number of virtual clients from 1 to 10,000. Each client sends 10 packets/s to the tested server for 60 s. The performance measurement module executes three iterations for each client transmission. If the setting for the number of clients is changed, the platform is given 60 s to cool down before continuing with the next step.

### 6.2. Power Consumption and Response Time with Resource Management

An IoT device runs its sensor device when it receives a request for sensor data. However, when the data request period increases, the IoT device must continuously operate its sensor device to measure sensing data. This causes an increase in power consumption of the IoT device. Most sensor devices do not need to update their sensing data, because most environmental data are stable and do not frequently change; it is therefore inefficient to continuously measure similar sensing data. 

MinT manages the measured data through efficient operation of the sensor devices, and defines two measurement methods to reduce the repetition of the sensor tasks, and therefore the power consumption. Firstly, MinT can adjust the measurement period of the sensor device; it stores the measured data of each sensor device into its resource storage for each cycle, and gives this to other IoT devices or application services. We therefore examine the power consumption according to the measurement interval. Using this, we can identify the optimal cycle for measuring sensor data. 

**Figure 14:**
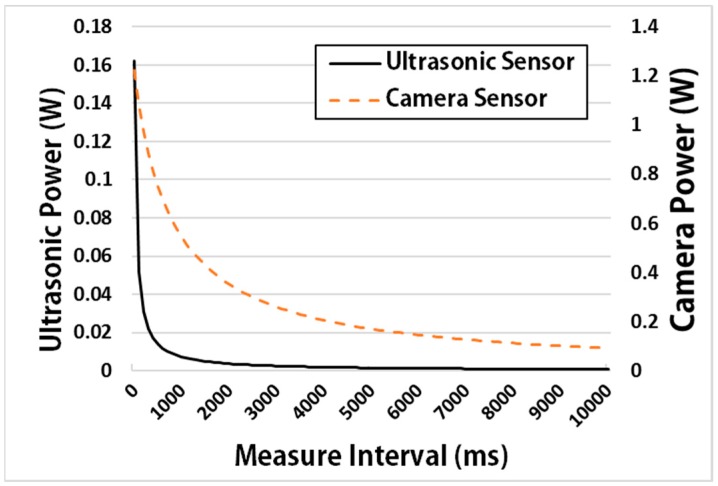
Power (Watt) of an ultrasonic sensor and camera sensor according to measure interval.

Figure 14 shows the power consumption according to the measurement interval of an ultrasonic sensor device. The ultrasonic sensor takes about 46 ms to measure the distance from it. It therefore consumes 0.16 W while it continuously measures the distance at an interval of 46 ms. In addition, Camera sensor takes about 832 ms to take a picture from it. It consumes 1.2 W while it continuously takes picture at an interval 832 ms. Figure 14 shows that the power consumption rate decreases sharply as the measurement interval increases.

This experiment has demonstrated that the proposed method can reduce the power consumption of an IoT device. In this paper, only an ultrasonic sensor and camera sensor were tested; other sensors may have different power consumption values to these sensors. However, we believe that other sensor devices will show similar results, because proposed method can adjust the measurement period of the sensor device and physically limits power consumption of the sensor device while not measuring data.

**Figure 15:**
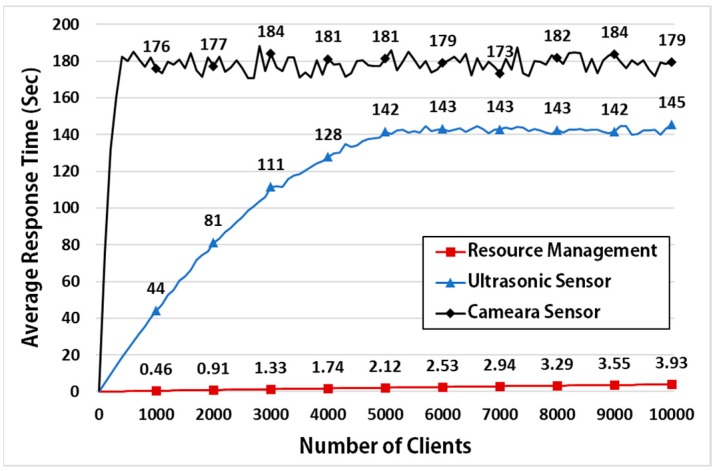
Response time of IoT device using ultrasonic and camera sensor.

Figure 15 shows the average response time of an IoT device with an ultrasonic sensor, relative to the number of clients. In our experiment, the server was tested with up to 10,000 clients and the request cycle set to 100 ms. Because the ultrasonic and camera sensor take a minimum of 46 ms and 832 ms to measure the distance, the results shows that the average response time increases rapidly as the number of clients increases (Figure 15). However, because MinT uses resource management to control the sensor device and to manage the sensing or network data, it can respond directly with the data stored in the resource storage. Therefore, the results show that the average response time is a maximum of about 3.9 s.

Some sensor data cannot be set to a longer measurement interval due to the data specifications. However, more efficient response times and power consumption can be achieved by setting the measurement period, because most environmental data are stable and do not frequently change.

### 6.3. Throughputs in IoT Middlewares

IoT devices must rapidly process a lot of requests that are received from other IoT devices. If IoT devices cannot process all requests, and message transmission is delayed or lost sequentially, responses to requests cannot be sent on time. The processing delays impact all connected IoT devices, this occurs network latancy and packet loss. 

**Figure 16:**
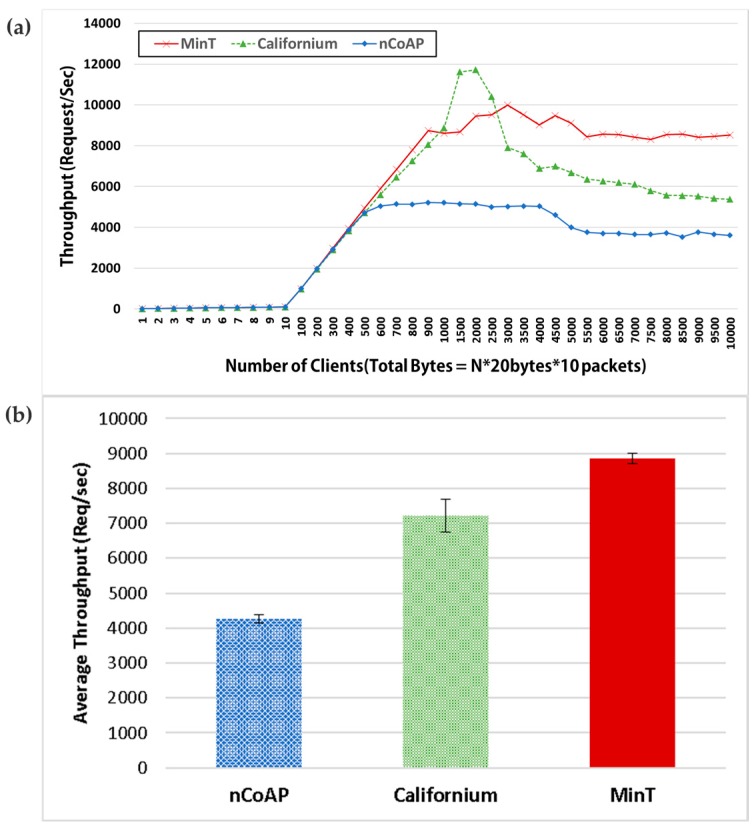
(a) Throughput and (b) average throughput with MinT, Californium, and nCoAP(SE).

Figure 16 shows the throughput characteristics achieved with MinT, Californium, and nCoAP in terms of total throughput and average throughput. Californium and nCoAP are middleware for message processing and resource management targeting back-end services and stronger IoT devices based on CoAP. Efficient and rapid message processing can increase efficiency of power consumption, reliability and scalability in the IoT network environment. In this paper, therefore, we propose the Interaction Layer of MinT for rapid message processing and evaluate its performance through comparison with Californium and nCoAP. 

In this experiment, the number of clients varies between 1 and 10,000. This figure represents the number of packets for a 60-s time period at each stage. As shown in the Figure 16, MinT and Californium produce the same throughputs up to 1000 clients. Beyond that point, Californium shows a significant increase in throughput in the interval between 1000 and 1500 clients. Beyond the 1500-client mark, the throughput of Californium begins to decrease sharply. This is owing to the fact that in Californium, when the number of requests reaches the point where the processing speed of the server becomes overmatched, the working queue volume increases significantly. 

The design of MinT prioritizes resource utilization of the system into the Interaction Layer, support the multi-threading in Interaction Layer to efficiently process requests according to performance of hardware platforms and simplifies the processing procedures for client requests. As a result, even if the number of clients simultaneously accessing the IoT network increases, MinT shows better performance compared to Californium. The nCoAP demonstrates an average throughput of 4500 requests/s, which is significantly lower when compared to the throughput numbers demonstrated by MinT and Californium. 

**Figure 17:**
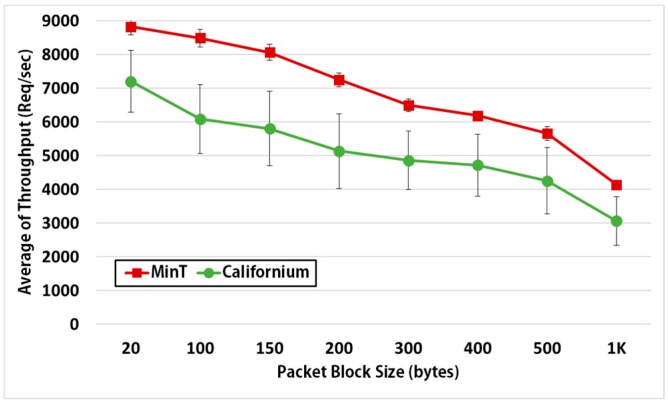
Average throughput for various packet block sizes in MinT and Californium (SE).

Figure 17 shows the variation of average throughput according to the packet block size. As the packet block size increases, the processing throughput of IoT device may decrease because the throughput of the IoT device and network bandwidth increase. Therefore, we conducted this experiment to test the throughput of IoT device according to the size of the requested data. To test this figure, we changed only the packet block size by repeating the same experiment as Figure 16. 

On average, MinT provides a performance increase of 25% compared to Californium across all the block sizes. In addition, if each thing exchanges the packet included many information at one swoop such as Discovery, the packet sizes might be 100~200 bytes. In this range, MinT demonstrates an average performance improvement of 28% compared to Californium. The performance of nCoAP is not a part of the performance comparisons owing to the fact that it showed the lowest performance of the three platforms.

### 6.4. Performance on Various Hardware Platforms

**Figure 18:**
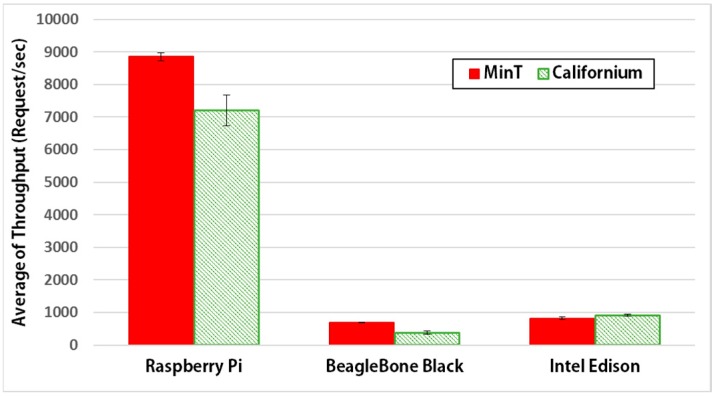
Average throughput performance on various hardware platforms for MinT and Californium (SE).

Figure 18 shows the average throughput performance on various hardware platforms for MinT and Californium. When compared to Californium, MinT shows about 19% better performance on the Raspberry Pi 3 and about 45% better on BeagleBone Black. However, MinT demonstrated about a 5% worse throughput on the Intel Edison, compared to Californium. Given the fact that both the BeagleBone Black and Intel Edison are low-end hardware platforms with low throughputs, the inferior performance of MinT on the Intel Edison need not be regarded to be of much significance. The experimental results show that MinT has the best performance on hardware platforms. As a result, we demonstrate that MinT is an efficient IoT middleware that can be utilized to achieve low transmission delays and enhanced battery life in an IoT environment. 

## 7. Conclusions

In this paper we have proposed a distributed IoT middleware solution called MinT, aimed at firstly addressing the problems of existing IoT middleware platforms, and secondly fulfilling other IoT middleware requirements. First, we designed the sensor abstract layer, which provides integrated operations for the various sensors and network devices. Because the SAL provides a powerful suite of APIs for developers, they can easily build and maintain IoT based applications. The abstracting nature of the SAL also allows MinT to enhance the re-usability of device drivers on other hardware or software platforms.

Second, we proposed resource management that helps to manage both the local data measured by sensor devices, and the network data received from other IoT devices. The Resource Manager in MinT defines two resource storages and sensor measurement modules to aim at reducing unnecessary sensor device behavior and reduce unnecessary IoT device processing. These allows MinT to enhanced the efficiency of use of the sensor device. Notably, this reduced the response time and power consumption of an IoT device. MinT controls the sensor device and manages the sensing or network data, it can respond directly with the data stored in the local resource storage. Therefore, the experimental results demonstrated an average response time decrease of 90% when resource management was used. In addition, MinT can adjust the measurement period of the sensor device and physically limits power consumption of the sensor device while not measuring data. Therefore, we can show the results of power consumption decreased by up to 68%.

Third, we designed the Connectivity Module to encourage efficient interaction between the IoT devices. This layer supports the network environments between variable network protocols via the Protocol Adapter module. To reduce the response times for requests passed between IoT devices, MinT also implements simplified networking technology and an optimized System Layer. It increases the CPU resource utilization of the Interaction Layer, and uses multi-threading for rapid request processing.

To evaluate the performance of MinT, we measured its job processing ability against existing IoT middleware platforms. The evaluations were performed to assess the throughput of various packet blocks. Experimental results show that MinT displayed performance improvements of 18% and 45% compared to Californium and nCoAP, respectively. Given the fact that in both two middlewares, when the number of requests reaches the point where the processing speed of the server becomes overmatched, the working queue volume increases significantly. Therefore, at this point, the throughput of both middlewares begins to decrease sharply. MinT responded more quickly than the other middleware types, showing that MinT-based IoT implementations can significantly reduce communication delays and power consumption in IoT devices.

Finally, because MinT provides open API libraries and the core specifications to operate IoT devices, developers can easily implement device drivers and service applications. The strong connectivity and networking functionality built into MinT also allows new IoT devices developed on MinT middleware to automatically link with surrounding IoT devices, and to retrieve and share information from cloud-based and social networks.

MinT also supports thread pooling to quickly process requests. Using a thread pool with a fixed number of threads has been shown to increase the efficiency of the CPU; however, this method can cause an unnecessary waste of resources. For example, when an IoT platform receives a number of requests that are smaller than its throughput, thread pooling does not offer any advantage and also wastes CPU resources. Creation of the threads also increases the platform’s memory usage, because multi-threading generally allocates an independent stack for each task. Therefore, a new method is required that efficiently controls memory and execution resources by directly adjusting the number of threads, so that requests from other devices are promptly processed. 

In future work we will propose an enhanced method of efficiently managing the thread pool, by adjusting the number of threads every cycle to regulate the performance of the IoT device. In particular, we will aim to improve the performance of the Interaction layer, which is responsible for analyzing, processing, and retransmitting the received packets. Eventually, MinT can reduce processing delays and power consumption of IoT devices in an IoT environment.

In addition, MinT currently only supports Linux, Windows or Android based IoT devices. This allows MinT to be used on most IoT devices currently in use. However, there are various other OS-based tiny IoT devices in the real world. We have not yet demonstrated the efficiency of MinT on devices using other OSs. Therefore, we will study tiny MinT for low power devices and demonstrate efficiency of tiny MinT. Tiny MinT is a lightweight middleware that can run on tiny OS (TinyOS, Contiki, RIOT, Arduino, etc.) for tiny IoT device.

## Figures and Tables

**Table 1:** Summary of the middlewares: supported functional requirements.

	Physically Distributed	Heterogeneous Devices	Spontaneous Interaction	Large-Scale Networks	Resource Management	Ease of Development
WSN and Cloud-centric IoT Middlewares						
Hydra	NS	S	NS	NS	MSO	S
UbiSoAP	NS	S	NS	S	MS	S
MOSDEN	NS	S	NS	IS	MS	S
Xively	NS	NS	NS	IS	MS	S
CarrIoTs	NS	NS	NS	IS	MS	IS
ThingSpeak	NS	S	S	IS	MS	S
Non-server based Distributed IoT Middlewares						
AndroidThings	S	S	S	S	NR	S
IoTivity	S	S	S	S	NR	IS
Californium	S	NS	S	S	NR	S
nCoAP	S	NS	NS	S	NR	IS

Legend: Not supported (NS). Supported (S). Insufficiency (IS). Management on-Server (MS). Ontology (O). Network Resource (NR).

**Table 2:** Main interface for hardware device development.

Function	Explanation
Init	Library load, Pin setting for hardware platform
Free	Free driver from memory
Command	Device command (Sensing, actuating, or function for operating device)
